# A multicenter study on two-stage transfer learning model for duct-dependent CHDs screening in fetal echocardiography

**DOI:** 10.1038/s41746-023-00883-y

**Published:** 2023-08-12

**Authors:** Jiajie Tang, Yongen Liang, Yuxuan Jiang, Jinrong Liu, Rui Zhang, Danping Huang, Chengcheng Pang, Chen Huang, Dongni Luo, Xue Zhou, Ruizhuo Li, Kanghui Zhang, Bingbing Xie, Lianting Hu, Fanfan Zhu, Huimin Xia, Long Lu, Hongying Wang

**Affiliations:** 1 Department of Medical Ultrasonics/Institute of Pediatrics, Guangzhou Women and Children’s Medical Center, Guangzhou Medical University, Guangzhou, China; 2 School of Information Management, Wuhan University, Wuhan, China; 3 Cardiovascular Pediatrics/Guangdong Cardiovascular Institute/Medical Big Data Center, Guangdong Provincial People’s Hospital, Guangzhou, China; 4 Department of Medical Ultrasonics/Shenzhen Longgang Maternal and Child Health Hospital, Shenzhen, China; 5 School of Medicine, Southern China University of Technology, Guangzhou, China; 6 Center for Healthcare Big Data Research, The Big Data Institute, Wuhan University, Wuhan, China; 7 School of Public Health, Wuhan University, Wuhan, China

**Keywords:** Echocardiography, Congenital heart defects

## Abstract

Duct-dependent congenital heart diseases (CHDs) are a serious form of CHD with a low detection rate, especially in underdeveloped countries and areas. Although existing studies have developed models for fetal heart structure identification, there is a lack of comprehensive evaluation of the long axis of the aorta. In this study, a total of 6698 images and 48 videos are collected to develop and test a two-stage deep transfer learning model named DDCHD-DenseNet for screening critical duct-dependent CHDs. The model achieves a sensitivity of 0.973, 0.843, 0.769, and 0.759, and a specificity of 0.985, 0.967, 0.956, and 0.759, respectively, on the four multicenter test sets. It is expected to be employed as a potential automatic screening tool for hierarchical care and computer-aided diagnosis. Our two-stage strategy effectively improves the robustness of the model and can be extended to screen for other fetal heart development defects.

## Introduction

CHD is one of the most common birth defects, with an estimated incidence of 3–12/1000 live births^1–3^. Duct-dependent CHDs are serious CHDs that rely on the postnatal patency of the ductus arteriosus to maintain adequate circulation. It is potentially life-threatening because infants may deteriorate when the ductus arteriosus closes after birth but present well during the fetal period^4,5^. Neonates with duct-dependent CHDs may suffer from severe cardiogenic shock, renal insufficiency, circulatory collapse, metabolic acidosis, and even death in the postnatal period soon^2,6–8^. This risk is greatest for defects with duct-dependent systemic circulation, notably aortic arch obstruction (including coarctation of the aorta (CoA) and interrupted aortic arch (IAA)), and is also significant in transposition of the great arteries (TGA)^4^. Therefore, it is promising to develop a low-cost and convenient screening method for duct-dependent CHDs.

Fetal ultrasound screening in mid-gestation allows clinicians to detect a large proportion of CHDs^9–12^, which is recommended for all pregnancies worldwide. A comprehensive assessment of the long axis of the aorta can supply critical diagnostic information and distinguish anomalous from normal structures since they are significantly malformed in the aortic view^4,5,13^. Fetuses identified with suspected aortic malformation during prenatal screening and subsequently referred to a tertiary care center for further examination and evaluation of peri-operative outcomes, prognosis can benefit from improved management^1,14^.

However, there was significant geographic variation in rates of prenatal detection. A study conducted in the United States indicates that the prenatal diagnosis rates of CHD on a state level range from 11.8 to 53.4%^15^, let alone, in underdeveloped countries and areas. As for critical duct-dependent CHDs including TGA, IAA and CoA, only 25–40% of fetuses with dextro-transposition of the great arteries (d-TGA) are accurately diagnosed in utero^2^; CoA is the most commonly missed fetal CHD diagnosis, with less than one-third of the cases being detected at prenatal screening^16–18^; and the prenatal detection rates of IAA are still only 50%^19^. Some researchers attribute the prenatal detection rates of regions to inadequate physicians' experience and insufficient quality of diagnostic images^2,3,20^. In this regard, artificial intelligence (AI) has become an essential technology to overcome these problems.

In recent years, AI has become a promising approach to assisting physicians^21^, which makes task-related decisions using expert knowledge gained from big data unlike doctors, who are influenced by personal experience^20^. The convolution neural network (CNN), one of the most promising methods in the field of AI, is used in obstetrical ultrasound because of its excellent performance in the analysis of medical images^22^. Quality control of ultrasound images is an essential step in assessing echocardiography. This task was automatically accomplished by Dong et al. utilizing the CNN approach in fetal ultrasound cardiac four-chamber view (4CV)^23^. The successful segmentation and classification of standard fetal cardiac views indicates that AI has a high potential for detecting structural malformations of the fetal heart^23–28^. Apart from image quality assessments and segmentation, recent studies have used CNN to identify fetal structures to timely find fetal abnormalities so that necessary action can be taken. Sundaresan et al. have proposed fully convolutional neural networks (FCNs) for detecting the fetal heart and identifying its views in the frames of antenatal ultrasound screening videos^25^. Gong et al. leveraged a new model, DGACNN, to decrease the influence of insufficient training datasets and training a robust model to accurately recognize the fetal CHDs during ultrasound images^29^. Rima Arnaout et al. trained an ensemble of neural networks for the identification of five recommended cardiac views (4CV, three-vessel view (3VV), left-ventricular outflow tract (LVOT), three-vessel trachea (3VT) and the abdomen (ABDO)) and the classification of normal and abnormal anatomy^30^. A more ambitious project using multi-CNN segments for the four standard heart views (4CV, 3VV/3VT, LVOT/RVOT) recognizes 24 objects, including four shapes of the fetal heart standard views, 17 objects of the heart chambers in each view, and three cases of congenital heart defects simultaneously^28^. Recent studies have developed models for anomaly identification using 4CV, 3VV, LVOT, RVOT, 3VT, and ABDO, but there is a lack of comprehensive evaluation of the long axis of the aorta, which is not conducive to rapid screening of critical duct-dependent CHDs such as IAA, TGA, or CoA; and the amount of data included was very limited, with only a few cases^30^.

Our study focused on utilizing the aortic arch view to screen for part of critical duct-dependent CHDs, including IAA, CoA and TGA. To achieve this, we developed a two-stage deep transfer learning method called DDCHD-DenseNet. We trained the algorithm on image datasets of different quality to address the challenges posed by non-standardization of ultrasound imaging. By leveraging transfer learning techniques, we improved the model’s ability to handle datasets with varying quality, enhancing its reliability and performance. Additionally, our study employed the Grad-CAM technique to identify structural abnormalities predicted by the model^31^. These predicted abnormalities showed a high correlation with the clinical manifestations of different diseases, contributing to the development of new artificial intelligence systems for distinguishing between various types of congenital heart diseases.

Our multicenter study utilizes a two-stage deep transfer learning model for screening critical duct-dependent CHDs from fetal echocardiography. The findings highlight the potential of this approach to improve the early detection and diagnosis of critical duct-dependent CHDs in fetuses, ultimately leading to better outcomes for infants with these conditions.

## Results

### Characteristics of the datasets

A total of 6698 images and 48 videos from the Guangzhou Women and Children’s Medical Center (GZMC), Shenzhen Longgang Maternal and Child Health Hospital (SZLG), and Guangdong Provincial People’s Hospital (GDPH) were used to develop and evaluate the deep learning system. In the training set, after image preprocessing and filtering by exclusion criteria, images were classified into criteria-specific datasets and general datasets. General datasets include 1929 images of duct-dependent CHD and 871 images without CHD. Criteria-specific datasets include 1823 images of duct-dependent CHD and 2089 images without CHD. In the testing set, three retrospective test sets from GZMC, SZLG and GDPH, and one prospective test set from GZMC were used to validate the screening performance of the model. The multicenter test set includes the above four test sets. Further information on datasets obtained from GZMC, SZLG, and GDPH, and ultrasound machines is summarized in Table 1.

**Table 1:** Characteristics of the internal datasets and external datasets.

Item		Internal datasets				External datasets	
Source		Retrospective (GZMC)			Prospective (GZMC)	SZLG	GDPH
Datasets		General datasets	Criteria-specific datasets		Multicenter test sets		
Use		Pre-train	Train	Test	Test	Test	Test
Normal (numbers)		1929	2089	200	91	46	54
Abnormal (numbers)	TGA	871	878	88	35	11	16
	CoA		509	42	31	13	27
	IAA		436	20	4	15	11
Ultrasound machine		GE Voluson E6/E8/E10, Philips iE33				GE Voluson E8/E10, Logqi E9-3, Aloka	GE Voluson E10, GE ViVi9, SSD-A(10)

In the process of model development, the Transfer Learning Group was compared with the General Group. The pre-training with general datasets and then transfer learning using criteria-specific datasets is named Transfer Learning Group. The group trained only with the criteria-specific dataset is the General Group.

### DDCHD-DenseNet outperforms other deep learning models in retrospective test set

**Fig. 1:**
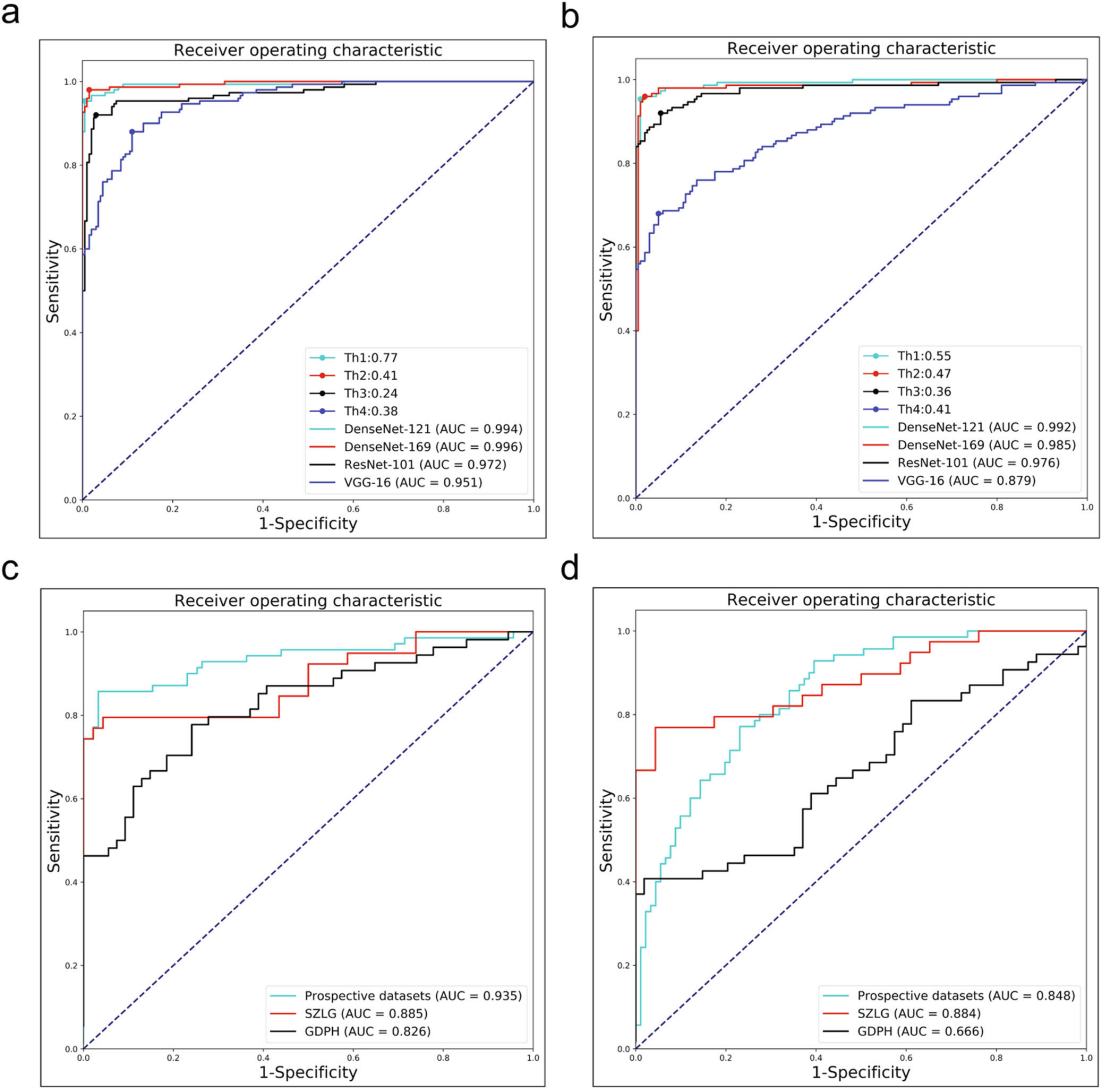
The performance of different deep learning algorithms in discerning duct-dependent CHD in four test datasets. **a** The performance of the Transfer learning group in screening duct-dependent CHD in retrospective datasets. **b** The performance of General Group in screening duct-dependent CHD in retrospective datasets. **c** The performance of DDCHD-DenseNet (Transfer Learning Group) in screening duct-dependent CHD in internal prospective datasets and two external datasets. **d** The performance of DenseNet-169 (General Group) in screening duct-dependent CHD in internal prospective datasets and two external datasets. Transfer Learning Group: developed the screening model using our two-stage transfer learning strategy. General Group: developed the screening model using the criteria-specific datasets. Th1, Th2, Th3, and Th4 correspond to the best threshold points of the DenseNet-121, DenseNet-169, ResNet-101, and VGG-16 models, respectively. SZLG Shenzhen Longgang Maternal and Child Health Hospital, GDPH Guangdong Provincial People’s Hospital. The distribution of predict scores related to the duct-dependent CHD determined by the Transfer Learning Group and the General Group in retrospective datasets.

In the Transfer Learning Group, DenseNet-169 yielded the following values in the retrospective test datasets: 0.996, 0.973, 0.985, and 0.977 corresponding to AUROC, sensitivity, specificity, and F1, respectively. The ROC curves of these algorithms in the retrospective test datasets are shown in Fig. 1a, with the AUROC, sensitivity, specificity, and F1 presented in Table 2, indicating that the optimal algorithm is the DenseNet-169. We named this transfer learning framework as DDCHD-DenseNet.

**Table 2:** Performance of Transfer Learning Group and General Group in the retrospective datasets.

	AUROC (95% CI)	Sensitivity (95% CI)	Specificity (95% CI)	F1
Transfer Learning Group				
DenseNet-169	0.996 (0.995–0.997)	0.973 (0.929–0.991)	0.985 (0.953–0.996)	0.977
DenseNet-121	0.994 (0.992–0.995)	0.947 (0.894–0.975)	0.995 (0.968–0.999)	0.969
ResNet-101	0.972 (0.971–0.976)	0.913 (0.853–0.951)	0.970 (0.933–0.988)	0.935
VGG-16	0.951 (0.947–0.953)	0.873 (0.807–0.920)	0.890 (0.836–0.928)	0.865
General Group				
DenseNet-169	0.985 (0.984–0.988)	0.953 (0.903–0.979)	0.980 (0.946–0.994)	0.963
DenseNet-121	0.992 (0.991–0.993)	0.947 (0.894–0.975)	0.990 (0.961–0.998)	0.966
ResNet-101	0.976 (0.973–0.979)	0.913 (0.853–0.951)	0.945 (0.901–0.971)	0.919
VGG-16	0.879 (0.872–0.885)	0.673 (0.591–0.746)	0.950 (0.907–0.974)	0.774

Data are metric value or metric value (95% CI).

**Fig. 2:**
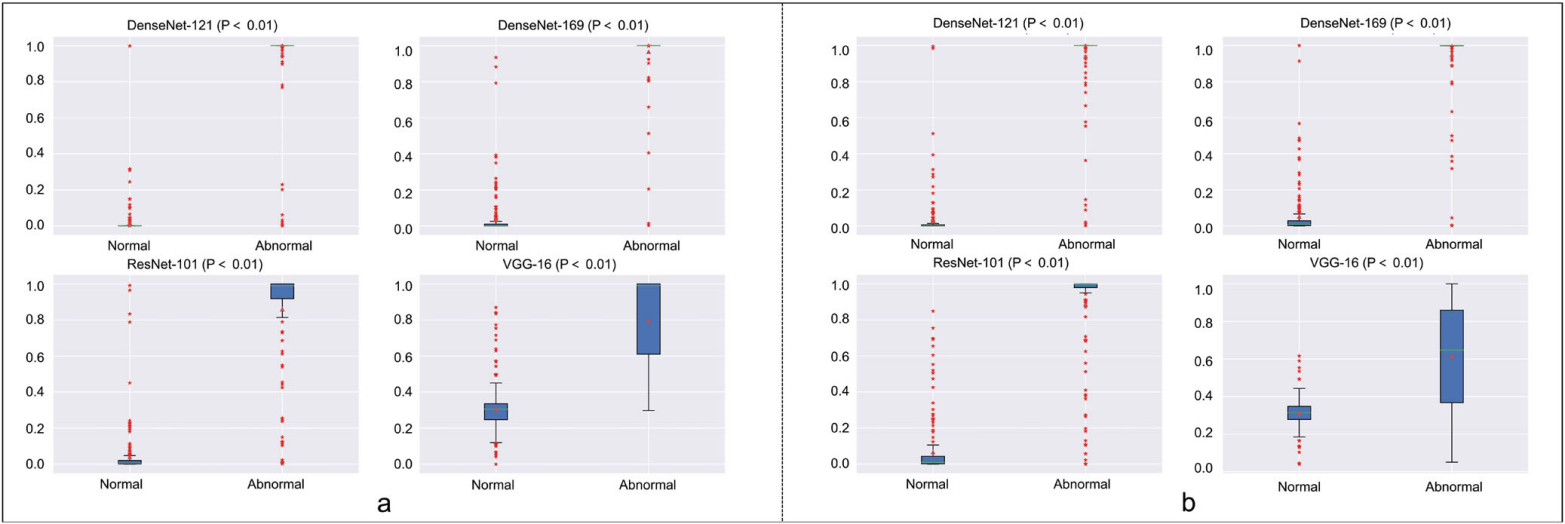
The distribution of predict scores related to the duct-dependent CHD determined by the Transfer Learning Group and the General Group in retrospective datasets. **a** Transfer Learning Group, **b** General Group. DDCHD-DenseNet uses the DensenNet-169 model architecture, and its performance is shown in (**a**) DenseNet-169. Risk scores (range 0–1) and confusion matrix predicted by the deep learning model for discerning fetal genetic diseases. Scores closer to 1 denote a higher probability of genetic diseases. The upper and lower bounds of the box refer to the 25th and 75th percentiles, and the line intersection in the box refers to the median. Whiskers refer to the full range of risk scores.

The distribution of predicted scores related to the duct-dependent CHD determined by the DDCHD-DenseNet in retrospective test sets is shown in Fig. 2a. With a threshold >0.41, the percentage of correctly predicted images in duct-dependent CHD was 98.9% (87/88) in TGA, 92.9% (39/42) in CoA, and 100% (20/20) in IAA. In addition, with a threshold of 0.41, the percentage of correctly predicted images in the normal fetal heart was 98.5% (197/200).

### Performance of the DDCHD-DenseNet in multicenter test sets

In multicenter test sets, two external test sets (SZLG and GDPH) and one internal test sets were used to evaluate the performance of DDCHD-DenseNet. The ROC curves of DDCHD-DenseNet in the three datasets are displayed in Fig. 1c, and the AUROC, sensitivity, specificity, and F1 are presented in Table 3, while the accuracy for screening duct-dependent CHD is displayed in Table 4.

**Table 3:** Performance of DDCHD-DenseNet and DenseNet-169 (General Group) in the multicenter test sets.

	AUROC (95% CI)	Sensitivity (95% CI)	Specificity (95% CI)	F1
DDCHD-DenseNet				
Internal prospective test sets	0.935 (0.934–0.942)	0.843 (0.732–0.915)	0.967 (0.900–0.991)	0.894
SZLG	0.885 (0.878–0.889)	0.769 (0.603–0.883)	0.956 (0.840–0.992)	0.845
GDPH	0.826 (0.820–0.832)	0.759 (0.621–0.861)	0.759 (0.621–0.861)	0.759
DenseNet-169 (General Group)				
Internal prospective test sets	0.848 (0.843–0.853)	0.757 (0.637–0.848)	0.769 (0.667–0.848)	0.736
SZLG	0.884 (0.883–0.894)	0.744 (0.576–0.864)	0.957 (0.840–0.992)	0.829
GDPH	0.666 (0.656–0.673)	0.389 (0.262–0.531)	0.981 (0.888–0.999)	0.553

Data are metric value or metric value (95% CI).

**Table 4:** Performance of DDCHD-DenseNet in screening TGA, CoA and IAA.

Accuracy	Retrospective datasets	Prospective datasets	SZLG datasets	GDPH datasets
Normal	98.5% (197/200)	96.7% (88/91)	95.7% (44/46)	75.9% (41/54)
TGA	98.9% (87/88)	91.4% (32/35)	63.6% (7/11)	68.8% (11/16)
CoA	92.9% (39/42)	74.2% (23/31)	100% (13/13)	74.1% (20/27)
IAA	100.0% (20/20)	100.0% (4/4)	66.7% (10/15)	90.9% (10/11)

In the internal prospective datasets, DDCHD-DenseNet achieved an AUC of 0.935, a sensitivity of 0.843, and a specificity of 0.967 in duct-dependent CHD screening. The percentage of correctly predicted images in duct-dependent CHD was 91.4% (32/35) in TGA, 74.2% (23/31) in CoA, and 100% (4/4) in IAA. In addition, the percentage of correctly predicted images in the normal fetal heart was 96.7% (88/91).

In the SZLG and GDPH datasets, DDCHD-DenseNet achieved AUCs of 0.885 and 0.826, sensitivity of 0.769 and 0.759, and specificity of 0.956 and 0.759 in duct-dependent CHD screening, respectively. In SZLG datasets, the screening accuracies of TGA, CoA, and IAA were 63.6% (7/11), 100% (13/13), and 66.7% (10/15), respectively. And in GDPH datasets, the screening accuracies in duct-dependent CHD were 68.8% (11/16) in TGA, 74.1% (20/27) in CoA, and 90.9% (10/11) in IAA.

### Transfer learning (PT-CHD weights) improves model performance on multicenter test sets

In retrospective test datasets, Transfer Learning Group has a slight improvement over the model performance of General Group. In DenseNet-169, the number of missed diagnoses in positive cases decreased from 7 to 4, and the number of misdiagnoses in negative cases decreased from 4 to 3. It is worth mentioning that the ROC of VGG-16 improved from 0.879 to 0.951 after transfer learning, which indicates that the deep network can learn more useful features when driven by data. The detailed information, including ROC, AUROC, accuracy, sensitivity, specificity, prediction scores, and F1 value of screening duct-dependent CHD are shown in Figs. 1 and 2 and Table 2.

The details on the performance of the General Group (DenseNet-169) and Transfer Learning Group (DDCHD-DenseNet) in the multicenter test sets are shown in Table 3 and Fig. 1c, d. Compared to the DDCHD-DenseNet, the AUC from General Group (DenseNet-169) is as low as 0.848 in the prospective test datasets, 0.884 in the SZLG datasets, and 0.666 in the GDPH datasets, respectively. It represents a significant reduction in the performance of screening for duct-dependent CHD in the General Group which indicates that the PT-CHD (weight) improves model performance on multicenter test sets.

We also conducted a reverse experiment, first training criteria-specific datasets, and then training general datasets to reveal that such transfer learning methods can cause negative transfer. The experimental results are shown in Supplementary Fig. 2 and Supplementary Table 1.

### DDCHD-DenseNet detects abnormal in aortic arch view illustrated by heatmap

**Fig. 3:**
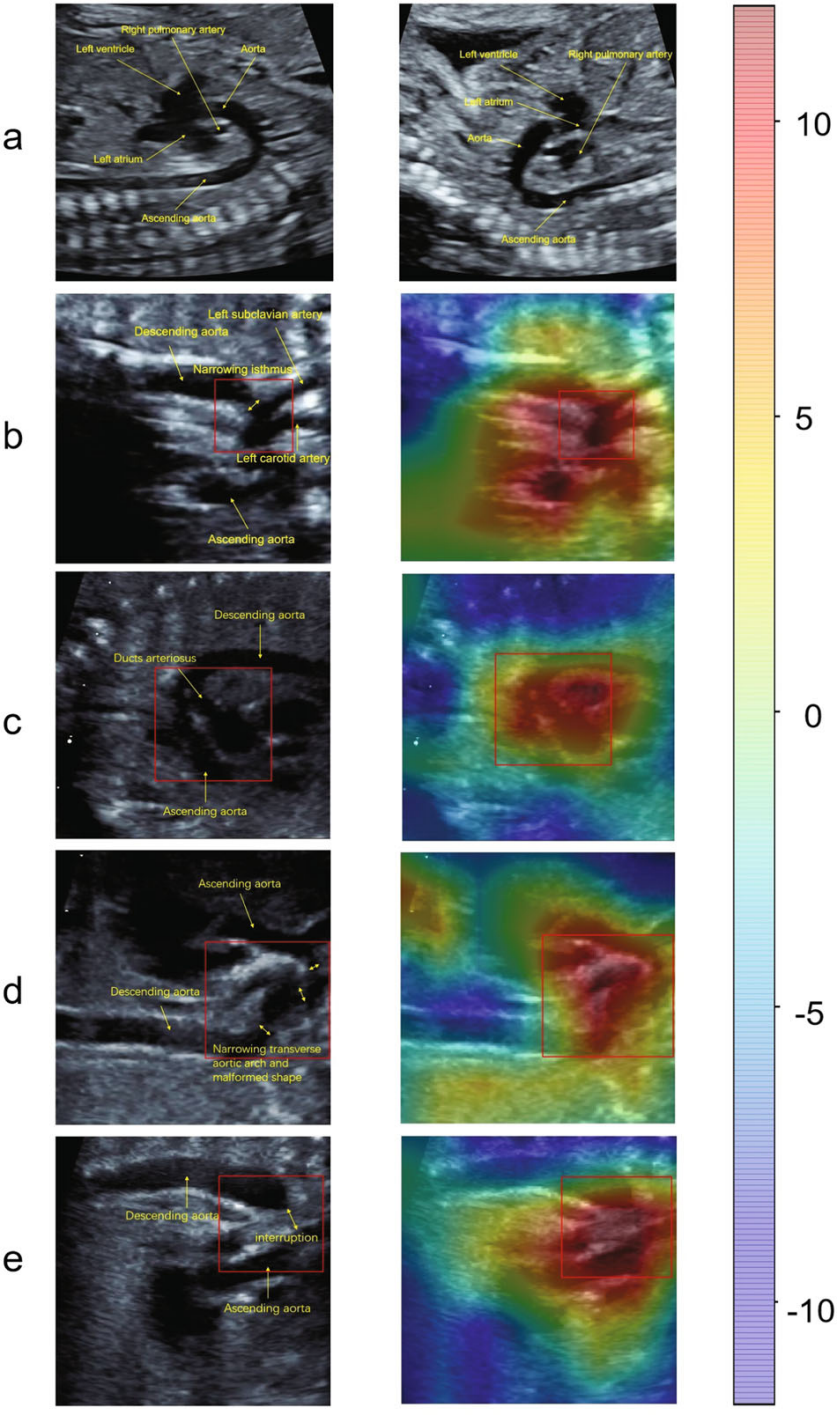
Example of images of duct-dependent CHDs and normal fetal hearts and corresponding heatmaps from DDCHD-DenseNet. The heatmap illustrates the importance of local areas within the image for being classified as duct-dependent CHDs, and the importance values are shown in the bar chart. The color red indicates higher importance, while the color blue indicates lower importance. The yellow words point to the anatomical structures and malformation locations. The red box represents the main deformity location. **a** The normal fetal heart. **b** Narrowing aortic isthmus in CoA. **c** The aorta and ductus arteriosus are parallel in the long axis view of the aortic arch in TGA. **d** Malformed shape and narrowing transverse aortic arch in another fetus of CoA. **e** Interruption between ascending aorta and descending aorta in IAA.

To investigate the interpretability of the DDCHD-DenseNet in screening duct-dependent CHD, heatmaps were created to visualize the regions that contributed most to the model’s decisions (Fig. 3). The importance score is scaled between −10 and 10, where a higher number indicates that the area is of higher importance for classifying the image as consistent with genetic diseases. During the image classification, we found that DDCHD-DenseNet detects abnormalities in the aortic arch view. In CoA, a narrowing occurs at the aortic isthmus (distal to the left subclavian artery) in Fig. 3b. In Fig. 3d, there is not only narrowing in the whole transverse aortic arch but also a represented malformed shape. The red region of the heatmap covers the whole transverse aortic arch in both of the above two pictures. In TGA, the aorta and duct artery do not cross but rather are seen coursing parallel to each other in the aorta sagittal view, and they are co-connected to the descending aorta. The red region of the heat map focuses on the parallel arteries and shape “Y” confluence, which consists of the aortic isthmus, ductus arteriosus, and the descending aorta. In IAA, there is a lack of continuity between the ascending aorta and the descending thoracic aorta, and the red region of the heat map overlapped the location of the interruption. From the output, we can see that the model perfectly focuses on the key areas of the image.

### DDCHD-DenseNet’s performance approaches that of senior sonographers

**Fig. 4:**
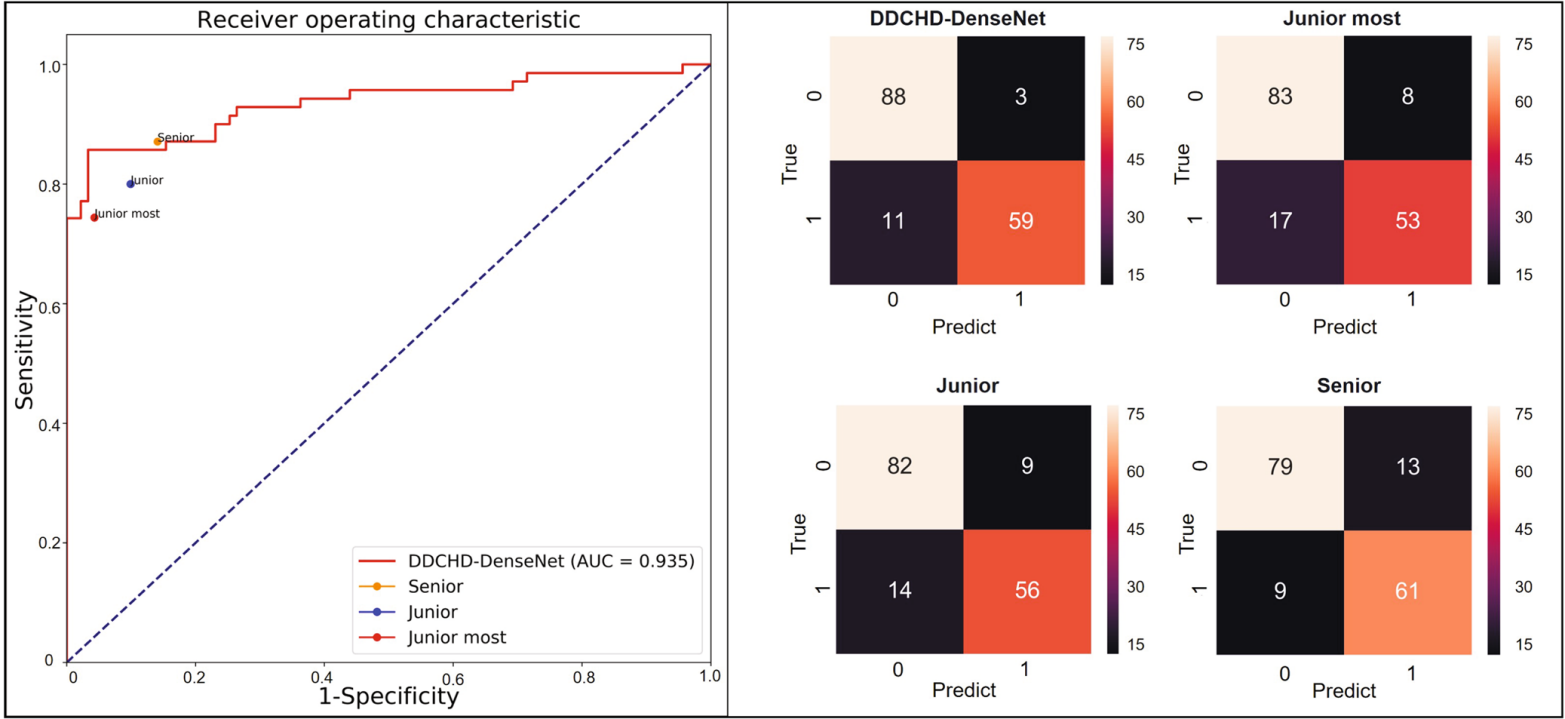
The ROC curves of the DDCHD-DenseNet and confusion matrix for screening for duct-dependent CHDs in prospective datasets and two external datasets with three levels of sonographer performance (junior-most, junior, and senior) to compare. In the confusion matrix, the horizontal coordinate is the predicted label, and the vertical coordinate is the true label. The distribution of predict scores related to the duct-dependent CHD determined by the Transfer Learning Group and the General Group in retrospective datasets.

We compared DDCHD-DenseNet’s performance in distinguishing a normal from an abnormal aortic arch view with that of sonographers of different seniorities in prospective datasets (Table 5 and Fig. 4). The accuracies of the junior-most and junior sonographers were lower compared with those of the DDCHD-DenseNet model in screening the images for duct-dependent CHD, i.e., the sensitivity of 0.744 by the junior-most, 0.800 by the junior, and 0.843 by DDCHD-DenseNet. The senior sonographers had more than 10 years of experience and achieved the best sensitivity of 0.871 and specificity of 0.859. Therefore, DDCHD-DenseNet demonstrated superior performance approaching that of senior sonographers in distinguishing normal from duct-dependent CHD via the screening aortic arch view.

**Table 5:** The screening performance of the DDCHD-DenseNet and sonographers with different seniorities.

	Sensitivity (95% CI)	Specificity (95% CI)	Accuracy				F1
			Normal	TGA	CoA	IAA	
DDCHD-DenseNet	0.843 (0.732–0.915)	0.967 (0.900–0.991)	96.7% (88/91)	91.4% (32/35)	74.2% (23/31)	100.0% (4/4)	0.912
Junior-most	0.744 (0.576–0.864)	0.957 (0.840–0.992)	91.2% (83/91)	94.3% (33/35)	51.6% (16/31)	100.0% (4/4)	0.869
Junior	0.800 (0.684–0.883)	0.901 (0.816-0.951)	90.1% (82/91)	94.3% (33/35)	61.3% (19/31)	100.0% (4/4)	0.877
Senior	0.871 (0.765–0.936)	0.859 (0.767–0.920)	86.8% (79/91)	94.3% (33/35)	77.4% (24/31)	100.0% (4/4)	0.878

Data are metric value or metric value (95% CI).

Among the duct-dependent CHDs judged as negative cases, CoA has the highest rate of missed diagnosis. The missed diagnosis rates for CoA were 22.6% (7/31) for the senior sonographers, 25.8% (8/31) for DDCHD-DenseNet, 38.7% (12/31) for the junior sonographers, and 48.4% (15/31) for the junior-most sonographers.

## Discussion

We developed a deep learning framework named DDCHD-DenseNet to detect malformation of the aorta on fetal ultrasound images that show a performance approaching that of the senior sonographers. In internal prospective test sets (GZMC) and the two external test datasets (SZLG and GDPH) consisting of fetal ultrasound images, the AUC for screening duct-dependent CHD is 0.935 in the prospective test sets, 0.885 in the SZLG test sets, and 0.826 in the GDPH test sets, respectively. In addition, the heatmaps from our system highlight the malformation regions of the heart on fetal ultrasound images.

Our study use deep learning techniques to screen for duct-dependent CHD in fetal echocardiography. In clinical practice, the aortic arch view is an important view in fetal heart examination, and this view contains important diagnostic information for these types of duct-dependent diseases. We developed DDCHD-DenseNet using aortic arch views, capable of distinguishing a normal aortic arch from the three major types of duct-dependent CHD, including TGA, CoA, and IAA. The model learns the morphological features of a normal aorta arch, and indicates its potential to screen for other diseases in these views. The deep learning-based approach enables us to screen for these types of congenital heart diseases dependent on arterial catheters faster and more accurately, reducing the rate of missed diagnoses. The DDCHD-DenseNet we developed is expected to be a low-cost, non-invasive, robust, and deployable prenatal screening strategy for duct-dependent CHDs, and can be extended to screen for other fetal heart development defects.

In the deep learning task based on ultrasound images, only data that the physician considers qualified is generally incorporated, but other data do not mean that they cannot provide valid clinical information. They can still provide some knowledge of the disease. Medical image data has the characteristics of small samples, and there is a shortage of high-quality image data, which can effectively utilize medical resources through transfer learning. Transfer learning can effectively utilize medical resources by leveraging existing knowledge to learn new knowledge. The core goal of transfer learning is to find the similarity between existing knowledge and the new knowledge. Images with low standardization and lower quality can provide preliminary disease knowledge in advance. These knowledge points are certainly helpful in disease screening and can enhance the model’s generalization, making them consistent with the concept of transfer learning. In this study, we first use lower-quality images (general datasets) for training to obtain the weight parameters, and then apply transfer learning techniques to train on the criteria-specific datasets. Comparative experiments on external test sets show that this method improves the robustness of the model.

The performance of DDCHD-DenseNet experienced only a slight decline when screening for duct-dependent CHD in prospective datasets from GZMC. The reason is that the majority of images in our center (GZMC) are obtained from fetal echocardiography, which has a relatively consistent cardiac magnification ratio. Some cases in SZLG are derived from obstetric screening, and the image magnification ratio of GDPH is insufficient, resulting in lower image quality. In a wide range of clinical applications, our DDCHD-DenseNet framework significantly improved the model’s robustness, especially by increasing the AUC values from 0.666 to 0.826 in GDPH and from 0.848 to 0.935 in prospective datasets from GZMC (internal prospective test sets).

Our two-stage transfer learning framework innovatively discovered that weight parameters obtained from general datasets for transfer learning can result in improved model performance in the case of limited medical data. The uneven quality and standardization of the data became our main challenge during the development of our model and guided the direction of our technical improvement. Previously, with the increasing availability of digitally archived medical imaging datasets, many efforts have been catalyzed to develop deep learning models to support patient care^32^. However, lower image quality, particularly prominent in prenatal diagnosis using ultrasound images, remains a significant challenge for clinical applications^33^. Initially, we utilized transfer learning to initialize the model weights, enabling the deep learning model to learn a generalized representation of duct-dependent CHD and handle non-standard images to some extent. Next, the model learns specific anatomical structures from datasets specific to the criteria through a second phase of training. Without the implementation of this two-stage transfer learning framework, the deep learning model cannot achieve the level of accuracy achieved in screening duct-dependent CHD (Fig. 1).

We generated the heat map using Grad-CAM to observe where the DDCHD-DenseNet focused when screening for duct-dependent CHD. When the DDCHD-DenseNet correctly screened for duct-dependent CHD, it appeared to focus on malformations of the aorta. For example, it identified the parallel configuration of the aorta and ductus arteriosus, forming a shape resembling the letter "Y" in cases of TGA; the narrowing of the aortic isthmus in CoA; and the lack of continuity between the ascending aorta and the descending thoracic aorta in IAA. However, when the DDCHD-DenseNet misclassified images, it sometimes seemed to focus on areas outside the aorta or detect artifacts in the ultrasound images. This illustrates why image quality can impact the classification performance of the deep learning model.

In an experiment comparing the sonographer and the DDCHD-DenseNet, the senior sonographer demonstrated a sensitivity of 0.871 and specificity of 0.859, whereas the DDCHD-DenseNet achieved a sensitivity of 0.843 and specificity of 0.967. These results indicated that the performance of DDCHD-DenseNet in screening for normal fetal heart and duct-dependent CHD in the aorta view is comparable to that of the senior sonographer, with a lower false-positive rate, thus avoiding unnecessary examinations and treatment. As an assistant screening tool, the DDCHD-DenseNet model outperforms junior sonographers but approaches the performance of senior sonographers in distinguishing between normal cases and duct-dependent CHDs through the aortic arch view, compensating for some missed and misdiagnosed cases resulting from experience dependency and image quality. It is worth mentioning that both sonographers and DDCHD-DenseNet exhibited a high rate of missed diagnosis in screening for CoA. Due to the subtle nature of partial lesions in the aortic isthmus, sonographers lacking extensive work experience and profound clinical knowledge may overlook these minor malformations and classify the abnormal aortic arch as normal. However, DDCHD-DenseNet demonstrates higher accuracy than junior and junior-most sonographers in detecting normal cases and CoA, and its performance approaches that of senior sonographers. This effectively improves the detection rate of CoA across various medical conditions.

### Study limitation and future perspective

As a study to employ deep learning for screening duct-dependent CHD in fetal ultrasound images, there is certainly ample room for improvement. Firstly, we utilized a simple automated cropping process to obtain the region of interest (ROI). While this approach alleviates the burden of manual segmentation, it can result in lower ROI quality in some images, thereby affecting the screening performance of the model. Further research should be conducted to explore more efficient and accurate methods of automatic segmentation for screening fetuses with duct-dependent CHD. Secondly, our study focused solely on screening for duct-dependent CHD and did not involve disease diagnosis. In future studies, we aim to incorporate more cardiac views to facilitate disease diagnosis research. Thirdly, although we utilized multicenter data for testing, we did not utilize multicenter data during model development, this will be addressed in our future endeavors.

## Conclusion

In this study, we developed a deep learning model named DDCHD-DenseNet for screening duct-dependent CHDs across various medical conditions. Its performance on multicenter test sets is closely approaches that of a senior physician, making it a promising automatic screening tool for hierarchical care and computer-aided diagnosis.

## Methods

### Data acquisition

**Fig. 5:**
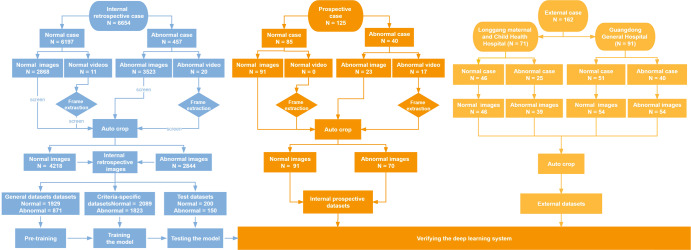
Flow chart for the development and evaluation of the deep learning system. SZLG Shenzhen Longgang Maternal and Child Health Hospital, GDPH Guangdong Provincial People’s Hospital, GZMC Guangzhou Women and Children’s Medical Center. The retrospective and prospective datasets were collected from GZMC, and external datasets were collected from SZLG and GDPH. Retrospective datasets from GZMC are used to develop models, and four datasets from three medical institutions were used for internal testing, prospective testing, and external testing.

This study is a multicenter research that included four datasets from three medical institutions. A total of 6698 images and 48 videos were collected from 6941 pregnancies at GZMC, SZLG and GDPH. Cases that lacked the aortic arch view were excluded. Detailed information on the datasets from each institution is presented in Table 1 and Fig. 5. Ultrasound examinations were performed using machines such as GE Voluson E6/E8/E10, GE ViVi9, Philips iE33, SSD-A(10), Logqi E9-3 and Aloka. The proportion of abnormal and normal group in different machines can be found in Supplementary Table 2.

Normal fetal hearts are defined as those that test negative for cardiac congenital malformations^1^. Duct-dependent CHDs in the aortic arch can manifest as any of the following lesions^2,3,9,17^: (1) In TGA, the aorta and ductus arteriosus do not cross but rather are seen coursing parallel to each other in the aorta sagittal view, and they are co-connected to the descending aorta; (2) CoA is defined as a localized narrowing of the aortic lumen; and (3) IAA is defined as a lack of continuity between the ascending and descending aorta. All prenatal ultrasonic diagnoses were confirmed through postnatal follow-up examinations, operations, or consensus comments provided by three senior sonographers with over 10 years of experience in prenatal care. The inclusion criteria were as follows: (1) Properly magnified images without noticeable acoustical shadow; (2) The heart being centered in the image, with clear cardiac features and no apparent obstruction or absence. Pregnancies that did not meet either of these image patterns were included in the general datasets, while qualified images were included in the criteria-specific datasets. Each image underwent two tiers of review by experienced sonographers for quality control. The operating procedure for sonographers can be found in Supplementary Note 1.

This study was approved by the GZMC institutional review board. Informed consents from external datasets (SZLG, GDPH) were exempted because of the retrospective nature of the study.

### Data preprocessing

First, we converted the images from DICOM format to JPG format and extracted the images from the video data (one image every 10 frames in the training set, one image every 50 frames in the test set). Second, we removed redundant backgrounds and borders, such as patient information, instrument settings, scales, and other background information, using an automated clipping procedure to obtain square graphical data. Third, we converted all images to grayscale. Next, we resized the images to a size of 256 × 256 pixels and normalized the pixel values to a range of 0 to 1. Fourth, we applied data augmentation techniques to increase the diversity of the datasets and alleviate the overfitting problem during the deep learning process. New samples were generated through simple transformations of the original images. We augmented the training dataset with distortion, zooming in, tilting, zooming out, and cropping. Further details can be found in Supplementary Fig. 1.

All preprocessing steps make use of open-source Python libraries: OpenCV, Scikit-Image, Augmentor and NumPy.

### The development process of DDCHD-DenseNet

**Fig. 6:**
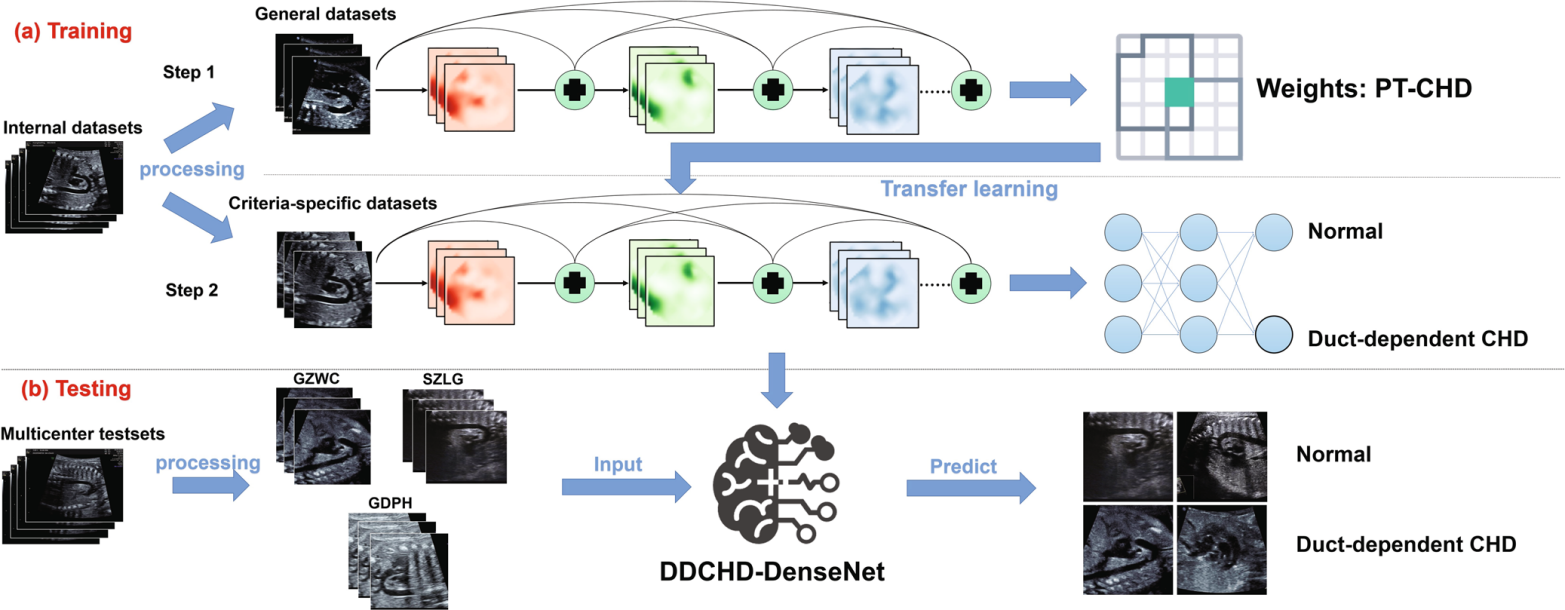
The development and evaluation process of the DDCHD-DenseNet. In step 1, the model weights PT-CHD are obtained by pre-training using general datasets. In step 2, transfer weights PT-CHD to learn the knowledge of congenital heart disease from criteria-specific datasets to develop DDCHD-DenseNet. In testing, four datasets from three medical institutions were used to evaluate the performance of the DDCHD-DenseNet in clinical applications.

Four classic deep learning algorithms, namely DenseNet-121, DenseNet-169, ResNet-101, and VGG-16, were employed in this study to develop DDCHD-DenseNet for screening duct-dependent CHD. The network architectures are described as follows, and the development process is illustrated in Fig. 6:

1. VGG: VGG is one of the most popular CNN models. By increasing the network’s depth, the model’s accuracy can be enhanced. It has been extensively utilized for extracting and analyzing features from medical images^34^.
2. DenseNet: by connecting preceding and subsequent layers, DenseNet can alleviate the problem of gradient disappearance, improve the efficiency of feature propagation and utilization, and reduce the number of parameters in the network. DenseNet has been employed for identifying keratitis and eyelid malignancies^35^.
3. ResNet: the residual neural network (ResNet) was proposed by He et al.^36^. The main advantage of ResNet is that it effectively addresses the issue of training deep neural networks. Recent studies have utilized ResNet to classify chest images of COVID-19 patients^37^.

At the start of the experiment, we conducted quality control on the ultrasound images based on the inclusion and exclusion criteria. We trained four screening models named the General Group using the criteria-specific datasets. To obtain the Transfer Learning Group, we initially trained four classical deep learning models using general datasets to obtain the pre-training weights (PT-CHD). Subsequently, we transferred the PT-CHD weights to the new deep learning model, which utilized the criteria-specific datasets for transfer learning. In the results, we compared the screening performances of General Group and Transfer Learning Group.

In all experiments, the Adam Optimizer was used to develop the model. The initial learning rate was set to 1e−4, the weight decay rate was 1e−7, the batch size was set to 64, the epoch was set to 100. During the training process, the validation parameter is set to 0.2, i.e., 20% of the training data is used as the tuning dataset, and the optimal model was saved through multiple training iterations.

### Statistical analysis

Six quantitative variables were utilized in the statistical analysis to evaluate the screening performance: accuracy, sensitivity, specificity, and F1 score. We also compared the screening performance of different models using the ROC curve and the AUC. Furthermore, ROC analysis was conducted using the outputs of the models on the retrospective datasets to determine the appropriate operating thresholds. Additionally, we employed the distribution of risk scores derived from deep learning in test sets for duct-dependent CHDs.

### Visualization of malformations in fetal heart

By incorporating a visualization layer to the deep learning model, Grad-CAM was employed to illustrate the system’s decision-making process. This technique generates a localization map that highlights important visual regions. The intensity of red indicates the relevance of the prediction. With the help of Grad-CAM, a heat map was generated to elucidate the reasoning behind DDCHD-DenseNet’s differentiation between normal fetal hearts and duct-dependent CHD.

### Competition of human and AI

Six sonographers with different levels of experience (two junior-most, two junior, and two senior) independently reviewed the same set of image datasets to distinguish between normal and abnormal aortic arch views in a separate test. The senior sonographers had more than 10 years of experience in conducting fetal anatomy scans and had performed more than 10,000 fetal ultrasound examinations. The junior sonographers had 5–10 years of experience and had conducted more than 5000 fetal scans. The junior-most sonographers, with 1–2 years of experience in fetal scans, had performed over 1000 fetal scans. It is important to note that in China, both the screening and diagnosis of fetal congenital heart disease are performed by ultrasonographers.

The prospective datasets used in this study were independent and unrelated to the training datasets. We selected these datasets for the purpose of conducting comparison experiments. The prospective dataset consisted of 91 negative cases and 70 positive cases, including 35 cases of TGA, 31 cases of CoA, and 4 cases of IAA. The datasets were divided into two groups and reviewed by sonographers at the same level. The results from each group were combined to obtain the final results for different levels. Each sonographer made judgments of normal and abnormal aortic arch views within a time limit of 3 min. To mitigate fatigue, sonographers were allowed to take a break every 30 min.

### Ethics approval and consent to participate

This study was approved by the Institutional Review Board of the Guangzhou Women and Children’s Medical Center (350b00, 2022). In the prospective data (GZMC), patients signed informed consent. But informed consents from external datasets (SZLG, GDPH) were exempted because of the retrospective data.

### Reporting summary

Further information on research design is available in the Nature Research Reporting Summary linked to this article.

### Supplementary information

Supplementary Material

Reporting Summary

## Data Availability

The data generated and/or analyzed during the current study are available upon reasonable request from the corresponding author. The data can be accessed only for research purposes. Researchers interested in using our data must provide a summary of the research they intend to conduct. The reviews will be completed within 2 weeks, and then a decision will be sent to the applicant. The data are not publicly available due to hospital regulatory restrictions.

## Code availability

The algorithms used in this study are all open-source code, including DenseNet, ResNet, and Grad-CAM. They can be accessed at the following links: https://github.com/liuzhuang13/DenseNet; https://github.com/ry/tensorflow-resnet; https://github.com/ramprs/grad-cam.
