# Supplementary material for: A multicenter study on two-stage transfer learning model for duct-dependent CHDs screening in fetal echocardiography
Source: NPJ Digit Med. 2023 Aug 12;6:143. doi: 10.1038/s41746-023-00883-y (PMC10423245; doi:10.1038/s41746-023-00883-y)
Supplement: Supplementary file 1 — Supplementary Material [file 41746_2023_883_MOESM1_ESM.docx]

## Supplementary Figure 1


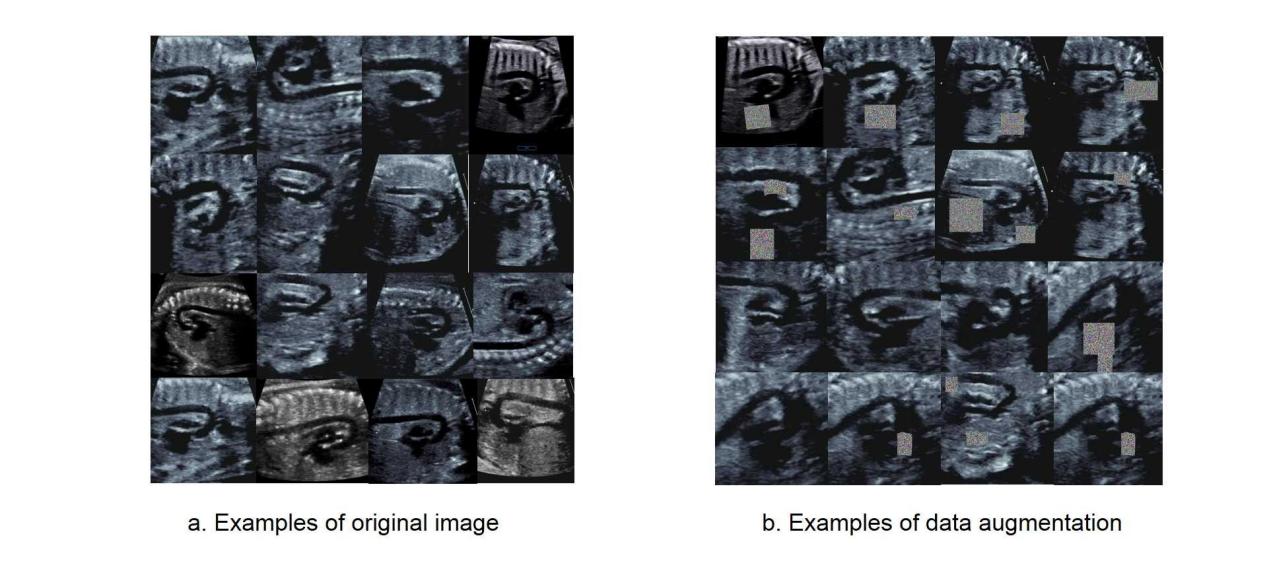


**Supplementary Figure 1**. a. Example of original. b. Example of data augmentation. The data augment used the “Augmentor” package (https://github.com/mdbloice/Augmentor), which can generate a fixed number of pictures by customization, and can freely combine the data augment methods. In this way, we only need to specify the data augmentation method and quantity, and it will automatically combine these methods. So we can maintain the sample balance of the training set in this way. We show the results of the data augmentation as follows:

**Supplementary Figure 2**


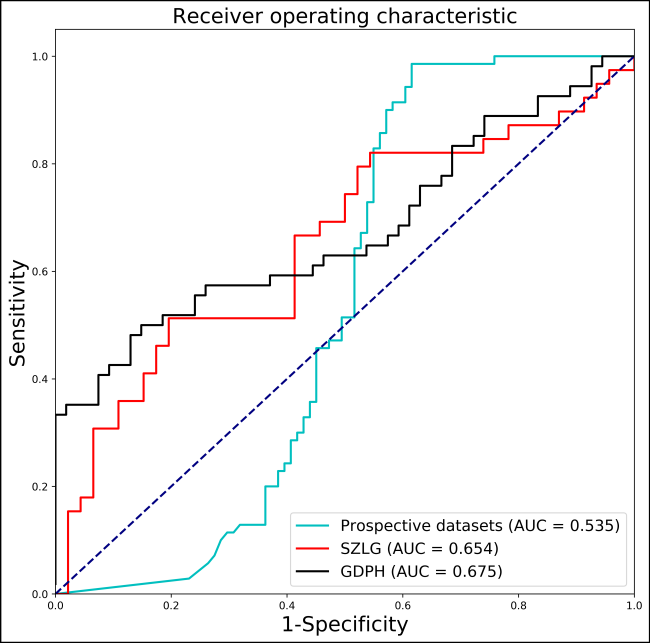


**Supplementary Figure 2**. We also conducted a reverse experiment, first training high-quality data sets, and then training low-quality data sets to reveal that such transfer learning methods can cause negative transfer. Model performance was evaluated using prospective datasets, SZLG and GDPH.

**Supplementary Table 1**

Sensitivity, specificity, and AUROC value of reverse experiments

|  | AUROC (95% CI) | Sensitivity (95% CI) | Specificity (95% CI) |
| --- | --- | --- | --- |
| Internal prospective testsets | 0.535 (0.526-0.545) | 0.971 (0.0.891-0.995) | 0.385 (0.286-0.493) |
| SZLG | 0.654 (0.639-0.657) | 0.487 (0.327-0.650) | 0.804 (0.656-0.901) |
| GDPH | 0.675 (0.662-0.679) | 0.463 (0.328-0.603) | 0.870 (0.745-0.942) |

**Supplementary Table 2**

The proportion of abnormal and normal group in different machines

| GZMC | Proportion(%) | | SZLG | Proportion(%) | | GDPH | Proportion(%) | |
| --- | --- | --- | --- | --- | --- | --- | --- | --- |
| Machine | Abnormal | Normal | Machine | Abnormal | Normal | Machine | Abnormal | Normal |
| GE Voluson E6 | 30 | 70 | GE Voluson E8 | 32 | 67 | GE Voluson E10 | 40 | 60 |
| GE Voluson E8 | 30 | 70 | GE Voluson E10 | 37 | 63 | GE ViVi9 | - | - |
| GE Voluson E10 | 30 | 70 | Loqig | 67 | 33 | SSD-A(10) | 66 | 34 |
| Philips iE33 | 40 | 60 | Philip | 100 | 0 |  |  |  |
|  |  |  | Aloka | 100 | 0 |  |  |  |

In GZMC, there is approximately 40% of abnormal group and 60% of normal group in Philips iE33. There is approximately 30% of abnormal group and 70% of normal group in GE Voluson E6/E8/E10. In SZLG, the proportion of abmormal group in GE Voluson E8, GE Voluson E10, Loqig, Philip and Aloka are respectively 32%, 37%, 67%, 100% and 100%. What needs explanation is that the cases collected from Philip and Aloka account a little part in abnormal cases. In GDPH, the proportion of abnormal group is approximately 40% in GE Voluson E10 and 66% in SSD-A(10). Images approximately came from Philips (65%), GE (30%), SSD-A(10), Loqig and Aloka (5%) ultrasound machines. The proportion of affected and control images in different machines is amply shown in Table A2.

**Supplementary Note 1**

Three experienced sonographers, each of whom had more than 10 years of fetal screening experience, were responsible for including the images that met the criteria. Two sonographers (D.H. and R.Z.) were responsible for the initial selection. Based on image quality, the images were divided into general datasets and criteria-specific datasets. Then, sonographer H.W. conducted a second check on the selected images to ensure that all of them met the inclusion criteria. The sonographers strictly followed the inclusion criteria, and if multiple images or frames from videos met the criteria, they randomly selected one of them.
