# Supplementary material for: A multicenter study on two-stage transfer learning model for duct-dependent CHDs screening in fetal echocardiography
Source: NPJ Digit Med. 2023 Aug 12;6:143. doi: 10.1038/s41746-023-00883-y (PMC10423245; doi:10.1038/s41746-023-00883-y)
Supplement: Supplementary file 2 — Reporting Summary [file 41746_2023_883_MOESM2_ESM.pdf]

nature portfolio

Corresponding author(s): Long Lu;Hongying Wang;Huimin Xia

Last updated by author(s): Jiajie Tang

Reporting Summary

Nature Portfolio wishes to improve the reproducibility of the work that we publish. This form provides structure for consistency and transparency in reporting. For further information on Nature Portfolio policies, see our Editorial Policies and the Editorial Policy Checklist.

Statistics

For all statistical analyses, confirm that the following items are present in the figure legend, table legend, main text, or Methods section.

n/a	Confirmed	
☐	☒	The exact sample size (*n*) for each experimental group/condition, given as a discrete number and unit of measurement
☐	☒	A statement on whether measurements were taken from distinct samples or whether the same sample was measured repeatedly
☐	☒	The statistical test(s) used AND whether they are one- or two-sided<br>*Only common tests should be described solely by name; describe more complex techniques in the Methods section.*
☐	☒	A description of all covariates tested
☐	☒	A description of any assumptions or corrections, such as tests of normality and adjustment for multiple comparisons
☐	☒	A full description of the statistical parameters including central tendency (e.g. means) or other basic estimates (e.g. regression coefficient) AND variation (e.g. standard deviation) or associated estimates of uncertainty (e.g. confidence intervals)
☐	☒	For null hypothesis testing, the test statistic (e.g. *F*, *t*, *r*) with confidence intervals, effect sizes, degrees of freedom and *P* value noted<br>*Give P values as exact values whenever suitable.*
☐	☒	For Bayesian analysis, information on the choice of priors and Markov chain Monte Carlo settings
☐	☒	For hierarchical and complex designs, identification of the appropriate level for tests and full reporting of outcomes
☐	☒	Estimates of effect sizes (e.g. Cohen's *d*, Pearson's *r*), indicating how they were calculated

Our web collection on statistics for biologists contains articles on many of the points above.

Software and code

Policy information about availability of computer code

Data collection	No software was used
Data analysis	python 3.8 ;keras 2.2 ;tensorflow2.0;numpy;pandas;opencv2

For manuscripts utilizing custom algorithms or software that are central to the research but not yet described in published literature, software must be made available to editors and reviewers. We strongly encourage code deposition in a community repository (e.g. GitHub). See the Nature Portfolio guidelines for submitting code & software for further information.

Data

Policy information about availability of data

All manuscripts must include a data availability statement. This statement should provide the following information, where applicable:

- Accession codes, unique identifiers, or web links for publicly available datasets
- A description of any restrictions on data availability
- For clinical datasets or third party data, please ensure that the statement adheres to our policy

The data generated and/or analyzed during the current study are available upon reasonable request from the corresponding author. The data can be accessed only for research purposes. Researchers interested in using our data must provide a summary of the research they intend to conduct. The reviews will be completed within 2 weeks, and then a decision will be sent to the applicant. The data are not publicly available due to hospital regulatory restrictions.

Human research participants

Policy information about studies involving human research participants and Sex and Gender in Research.

Reporting on sex and gender	Our research is based on women, so all participants are women and do not include gender related studies
Population characteristics	Female, aged 20-40
Recruitment	The participants in the experiment were all patients from our hospital who filled out informed consent forms before participating in the experiment
Ethics oversight	This study was approved by the Institutional Review Board of the Guangzhou Women and Children's Medical Center (350b00, 2022).

Note that full information on the approval of the study protocol must also be provided in the manuscript.

Field-specific reporting

Please select the one below that is the best fit for your research. If you are not sure, read the appropriate sections before making your selection.

☒ Life sciences ☐ Behavioural & social sciences ☐ Ecological, evolutionary & environmental sciences

For a reference copy of the document with all sections, see nature.com/documents/nr-reporting-summary-flat.pdf

Life sciences study design

All studies must disclose on these points even when the disclosure is negative.

Sample size	The samples were determined through the hospital's database, and we included patients diagnosed with this disease in our center over the past 10 years. Due to the limited number of patients, we can only include these cases for research.
Data exclusions	The inclusion criteria were as follows: 1) Images were properly magnified, with no discernible acoustical shadow; 2) The heart is in the center of the image, with distinct cardiac features and no obvious occlusion or absence. Pregnancies that did not match either of these two image patterns were included in the lower-quality datasets and the qualified images were included in the qualified datasets. All cases with missing clinical data were excluded, especially those without follow-up investigation results.
Replication	Conduct experiments again using the dataset, train deep learning models, and evaluate the effectiveness of the models. However, the training model is uncontrollable, so a large number of experiments are required to obtain reliable results.
Randomization	This information is not related to this study and does not involve human experiments. This study established a deep learning model using ultrasound images.
Blinding	The internal test set grouping in this study is randomly assigned, with one part trained for the model and the other part tested for the model. However, the external test set is fully included in the testing, and there is no grouping.

Reporting for specific materials, systems and methods

We require information from authors about some types of materials, experimental systems and methods used in many studies. Here, indicate whether each material, system or method listed is relevant to your study. If you are not sure if a list item applies to your research, read the appropriate section before selecting a response.

Materials & experimental systems

n/a	Involved in the study	
☒	☐	Antibodies
☒	☐	Eukaryotic cell lines
☒	☐	Palaeontology and archaeology
☒	☐	Animals and other organisms
☐	☒	Clinical data
☒	☐	Dual use research of concern

Methods

n/a	Involved in the study	
☒	☐	ChIP-seq
☒	☐	Flow cytometry
☒	☐	MRI-based neuroimaging

Clinical data

Policy information about clinical studies

All manuscripts should comply with the ICMJE guidelines for publication of clinical research and a completed CONSORT checklist must be included with all submissions.

Clinical trial registration	No clinical experiments were conducted, we only used ultrasound imaging
Study protocol	Retrospective and prospective collection of experimental data, development of deep learning models to achieve automatic diagnosis of diseases. Evaluate model performance using external test sets and visualize the model using interpretable methods. Finally, design a comparative experiment and compare it with the doctor.
Data collection	The data is sourced from the Women and Children's Medical Center in Guangzhou, Guangdong Province, China, and was collected from 2012 to 2022
Outcomes	The gold standard for data comes from the clinical diagnostic results of the hospital, and these results are recorded in follow-up (we can ensure the accuracy of the diagnostic results). The performance of deep learning models is reflected in the difference between real and predicted values. This article uses AUC, sensitivity, specificity, F1 value, etc. to measure these differences.
